# Simultaneous determination of two galangin metabolites from *Alpinia Officinarum* Hance in rat plasma by UF LC-MS/MS and its application in pharmacokinetics study

**DOI:** 10.7717/peerj.11041

**Published:** 2021-03-16

**Authors:** Rangru Liu, Hailong Li, Na Wei, Yinfeng Tan

**Affiliations:** 1 Hainan Provincial Key Laboratory of R&D of Tropical Herbs, School of Pharmacy, Hainan Medical University, Haikou, China; 2 Haikou Key Laboratory of Li Nationality Medicine, School of Pharmacy, Hainan Medical University, Haikou, China; 3 Key Laboratory of Tropical Diseases and Translational Medicine of the Ministry of Education, Hainan Provincial Key Laboratory of Tropical Medicine, Hainan Medical University, Haikou, Hainan, China

**Keywords:** *Alpinia Officinarum* Hance, Galangin, Galangin-3-o-β-D-glucuronic acid, Galangin-7-o-β-D-glucuronic acid, Plasma concentration, UFLC-MS/MS

## Abstract

Galangin has multiple pharmacological efficacies, such as anti-cancer, anti-inflammation and anti-oxidation. Galangin can be rapidly converted into glucuronidated metabolites in vivo. This study aimed to establish an UFLC-MS/MS analytical method to simultaneously determine the concentrations of two glucuronidated metabolites of galangin, galangin-3-O-β-D-glucuronic acid (GG-1) and galangin-7-O-β-D-glucuronic acid (GG-2) in rat plasma. After oral administration of galangal extract (0.3 g/kg), blood samples were collected from the orbital sinus, then treated by methanol precipitation and further gradient-eluted with Phenomenex Kinetex 2.6 µm XB-C18 column. The mass spectrometer was manipulated in the negative electrospray ionization (ESI) and selected multiple reaction monitoring (MRM) mode for the analytes. The precursor-to-product ion pairs applied for GG-1, GG-2 and chrysin (as the internal standard, IS) were m/z 445.2→269.0, 445.2→268.9 and 253.0→142.9, respectively. The results showed that the linear ranges for both GG-1 and GG-2 were 2.0–2000.0 ng/mL (*r*^2^ > 0.995). The inter- and intra-day precision were 89.3%–109.2%, RSD was less than 15%, and the repeatability was good. The recoveries of both metabolites and IS were over 89%, and matrix effect was within 15%. The validated analytical method was further applied to study the pharmacokinetic profiles of GG-1 and GG-2 in vivo. The pharmacokinetic parameters suggested that T_max_ of GG-1 was equivalent to that of GG-2, and MRT_0-t_, *t*_1/2_ of GG-2 were a little higher than those of GG-1. Importantly, AUC_0-t_ and C_max_ of GG-2 were almost twice as those of GG-1. In short, the validated UFLCMS/MS analytical method was feasible to simultaneously determine two galangin metabolites GG-1 and GG-2 in rat plasma and further analyze in vivo metabolism of galangin.

## Introduction

*Alpinia officinarum* Hance, known as galangal, is a traditional Chinese medicine, the rhizome part of which is often applied to treat epigastric cold and pain, stomach cold, vomiting and other diseases. Phytochemical studies have shown that galangal mainly contain flavonoids, diphenylheptane, phenylpropanol, volatile oil and other chemical components (Abubakar et al., 2018). In our previous study, we reported a simultaneous quantification method for 17 chemical components in galangal (Zhang et al., 2015). In addition, we optimized the extraction process of galangal and obtained galangal extract in which galangin content was up to 11.8% (Cheng et al., 2017; Cheng et al., 2015). As one main active component of galangal, galangin (3,5,7-trihydroxyflavone) has multiple biological activities, including anti-cancer (Ha et al., 2013; Su et al., 2013; Zhang et al., 2013), anti-oxidative (Choi & Kim, 2014; Shu et al., 2014), anti-inflammatory (Jung et al., 2014; Kim, Bae & Kim, 2013) , lipid-lowering (Jung et al., 2012; Kumar & Alagawadi, 2013), anti-microbial (Schnitzler et al., 2010), anti-viral (Cushnie & Lamb, 2006) and vasorelaxant effects (Morello et al., 2006).

**Figure 1:**
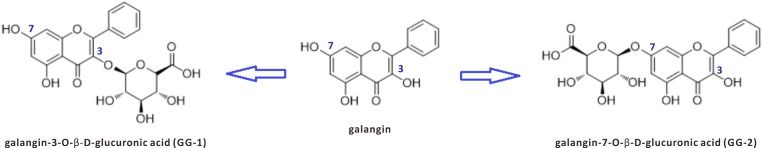
The chemical structures of galangin and its two metabolites GG-1 and GG-2.

However, even if they can be isolated from natural sources, most natural products cannot be directly used as a drug due to their low biological availability, low bioactivity and high toxicity and so on. Further finding the active metabolites from biological samples has proved to be an effective strategy in drug discovery. Our previous pharmacokinetic studies have indicated that galangin can be rapidly converted into glucuronidated metabolites after oral or intravenous administration in rats (Chen et al., 2015). Two galangin metabolites, galangin-3-O-β-D-glucuronic acid (GG-1) and galangin-7-O-β-D-glucuronic acid (GG-2), were isolated from rat urine that was collected after oral administration of galangin (Zhang et al., 2016). The chemical structures and relationships of galangin, GG-1 and GG-2 are shown in Fig. 1. It is worth notable that both metabolites, GG-1 and GG-2, possessed better lipid-lowering activities than galangin itself. Therefore, we intend to conduct a systematic study on galangin metabolites.

Although an analytical method based on ultra-performance liquid chromatography (UPLC) was established to determine galangin concentration in rat plasma by adopting diazepam as IS (Feng et al., 2015), there are no well-established analytical methods to quantitively determine two galangin metabolites GG-1 and GG-2 in vivo and much less their pharmacokinetic study. Hence, it is very necessary to develop an accurate, rapid and selective bioanalytical method for the determination of GG-1 and GG-2 in plasma. Ultra-fast liquid chromatographic coupled with electrospray ionization triple quadrupole tandem mass spectrometry (UFLC–MS/MS) technology provides a new analytical method for fast sample throughput and is appropriate for the analysis of the complex system of traditional Chinese medicine, especially for the low concentration components.

This work firstly established and validated an UFLC-MS/MS analytical method to simultaneously determine two galangin metabolites GG-1 and GG-2 in rat plasma, which was further successfully applied in pharmacokinetic study for GG-1 and GG-2 after oral administration of galangal extract, and provided a feasible in vivo analytical method for galangin metabolites.

## Materials & Methods

### Chemicals and reagents

GG-1 and GG-2 standards (the purity of both standards was over 97%) were separated from rat urine after oral administration of galangin according to previous reports and quantified by HPLC–PDA–MS (Zhang et al., 2016). As IS, chrysin (the purity: 98.0%) was separated from *A. oxyphylla* fruits by our laboratory and confirmed through MS and NMR analysis. HPLC-grade formic acid and methanol were bought from Aladdin Industrial Inc. (Shanghai, China). Double distilled water was obtained by the LabTower EDI15 system (Thermo Scientific, Waltham, MA, USA). Anesthetics isoflurane was purchased from Gene&I Corporation (Beijing, China). Galangal extract was prepared by our laboratory as the reported method (Cheng et al., 2017; Cheng et al., 2015). HPLC analysis results suggested that galangin content was determined to 11.8% in galangal extract and the other known components were Kaempferide 2.3%, DPHA 20.3%, DPHB 5.1% and DPHC 21.1%, respectively. The rest of the analytical reagents were commercially available.

### Experimental animals

Sprague Dawley (SD) rats (male, 200–250 g) were obtained from Changsha TianQing Biotechnology Co., Ltd. (Changsha, China; No.43006700006014). The rats were fed with free access to fodder and water at room temperature. The rats were adapted to the environment for a short time and experienced an overnight fasting period before oral administration. The experiment was approved by the animal ethics committee of Hainan Medical University (the approval number: 2019072107/HMU) and implemented according to the Guidance for Ethical Treatment of Laboratory Animals (The Ministry of Science and Technology of China, 2006).

### Drug administration and blood collection

Galangal extract was weighed precisely and suspended in water (with co-solvent Tween-80) to 30 mg/mL. Serial blood samples of approximately 0.2 mL were collected from orbital venous at 0, 0.0833, 0.25, 0.5, 1, 2, 4, 6, 8, 12, 24 h in five rats after a single oral administration of galangal extract (0.3 g/kg). The dose selection was referred to the previous study (Gong et al., 2018). After blood samples were collected, the rats were euthanized. After centrifugation, plasma fraction was obtained and preserved at −70 °C until analysis.

### Plasma samples preparation

IS-spiked methanol solution (150 μL, chrysin was 40 ng/mL) was added to plasma samples (60 μL). The mixture was vortexed vigorously for 10 min and centrifuged at 13,000 for 10 min. The upper supernatant was enriched under N_2_ via a TechneTM Sample Concentrator. Methanol (30 μL) was added to the concentrate, vortexed and centrifuged *ditto*, then 10 μL of the supernatant was used for LC-MS/MS analysis.

### Instruments and experimental conditions

LC-MS/MS system included a Shimadzu Prominence UFLC chromatographic system (Shimadzu, Kyoto, Japan) and API 4000+^TM^ triple quadrupole mass spectrometer (AB-SCIEX, Canada) equipped with an ESI source (AB-SCIEX, USA). Data collection was operated with AB-SCIEX Analyst software 3.0. Chromatographic separation was accomplished at 40 °C on a Phenomenex Kinetex 2.6 μm XB-C18 column (2.1 mm i.d. ×5 mm, 100 Å) with a guard column (0.5 μm, Upchurch, USA). The mobile phase was composed of water (contain 0.01% formic acid, A)-methanol (contain 0.01% formic acid, B), flow rate was 0.35 mL/min with a gradient elution: 0–0.01 min (1%B), 0.01–0.50 min (1%B), 0.51–4.0 min (40%B→50%B), 4.01–8.00 min (80%B→100%B), 8.01–10.00 min (1%B).

Mass spectrometer was manipulated in the negative ion and selected multiple reaction monitoring (MRM) mode for the analytes. MS/MS operating conditions were optimized by infusion of the standard solution (1 μg/mL) of each analyte and IS into the ESI source via a syringe pump (Harvard Apparatus, USA). The precursor-to-product ion pair and collision energy (CE) for quantification of the analytes as follows: m/z 445.2→269.0 (CE, −24 V), 445.2→268.9 (CE, −25 V), 253.0→142.9 (CE, −37 V) for GG-1, GG-2 and IS, respectively.

### Method validation

The method validation was operated referring to the Guidance for Industry Bio-analytical Method Validation (USFDA, 2018). Standard stock solutions of the analytes and IS were prepared by dissolving approximate 1mg of accurately weighted the analytes and IS in one mL of methanol. The 1 mg/mL the analytes stock solution was diluted with methanol to 20, 50, 100, 200, 1,000, 10,000 and 20,000 ng/mL series working concentration of solution. The working concentration of IS solution was 40 ng/mL.

#### Specificity and selectivity

The specificity of the method was investigated through comparing the chromatograms of six different blank plasma samples, blank plasma spiked with the analytes and IS at lower limit of quantification (LLOQ) concentration and plasma samples collected after oral administration of galangal extract (0.3 g/kg) in rats. The selectivity was evaluated by the peak interference of the analytes to IS.

#### Calibration curve and LLOQ

Seven standard calibration concentration levels (2, 5, 10, 20, 100, 1,000, 2,000 ng/mL) of plasma samples were prepared in quadruplicate by adding 6 uL of 20, 50, 100, 200, 1,000, 10,000 and 20,000 ng/mL series working concentration of standard solution to 54 uL of blank plasma and oscillation blend. The calibration curves were established by plotting peak area ratios (y) of each analyte to IS versus nominal concentrations (x) of least square linear regression analysis with a weighting factor 1/x. LLOQ was ruled as the lowest concentration that can be quantified on the condition of precision not exceed 20% and accuracy within ±  20%.

#### Precision and accuracy

The intra-day precision and accuracy were analyzed with three levels of quality control (QC) samples (*n* = 5) within the same day. The inter-day precision and accuracy were assessed with three batches QC samples on three consecutive days. The accuracy and precision must meet the criterions: the intra- and inter-day precision (RSD) must not more than 15% and accuracy should be less than ±  15%.

#### Extraction recovery and matrix effect

The extraction recovery and matrix effect were evaluated referring to the previous reported method by comparing the analysis results of three sample sets (Matuszewski, Constanzer & Chavez-Eng, 2003). The analytes were added in matrix component-free reconstitution solvent, in pre-extracted blank plasma and in blank plasma and then extracted for set 1, set 2 and set 3, respectively. The Extraction Recovery = A_set2_/A_set3_, Matrix Effect = A_set1_/A_set2_. Each batch of the samples contained three concentration levels and each level was performed in quintuplicate.

#### Stability

The stability of the analytes in plasma was evaluated at three QC levels in four storage conditions: (1) Three freeze–thaw cycles; (2) Short-term stability of plasma samples pre-extracted at room temperature; (3) After being prepared 8 h at auto-sampler; (4) 15 days at −20 °C. The stability (RSD) must be less than 15%.

#### Residual effect and dilution reliability

The quantitative upper limit sample (2,000 ng/mL) was injected for analysis immediately after the blank sample was analyzed, and the residue of the analytes and IS on the blank sample atlas was investigated. Six samples with the analyte concentration exceeding the quantitative upper limit were prepared with blank plasma and the dilution factor of 10 times was investigated.

### Application in pharmacokinetics study

Plasma samples were prepared from 5 male SD rats according to the methods as described in section “3. Drug Administration and Blood Collection”, and plasma concentrations of GG-1 and GG-2 were determined by the established method at each time point. To acquire pharmacokinetic parameters, the concentration-time curve was obtained by a non-compartmental method using DAS software package (version 3.2.8; Shanghai, China). The C_max_ and T_max_ were observed values with no interpolation. The area under concentration-time curve up to the last measured time point (AUC_0→*t*_) was calculated by the trapezoidal rule method. All data were indicated as the mean ±  SD.

## Results

### Method validation

#### Specificity and selectivity

**Figure 2:**
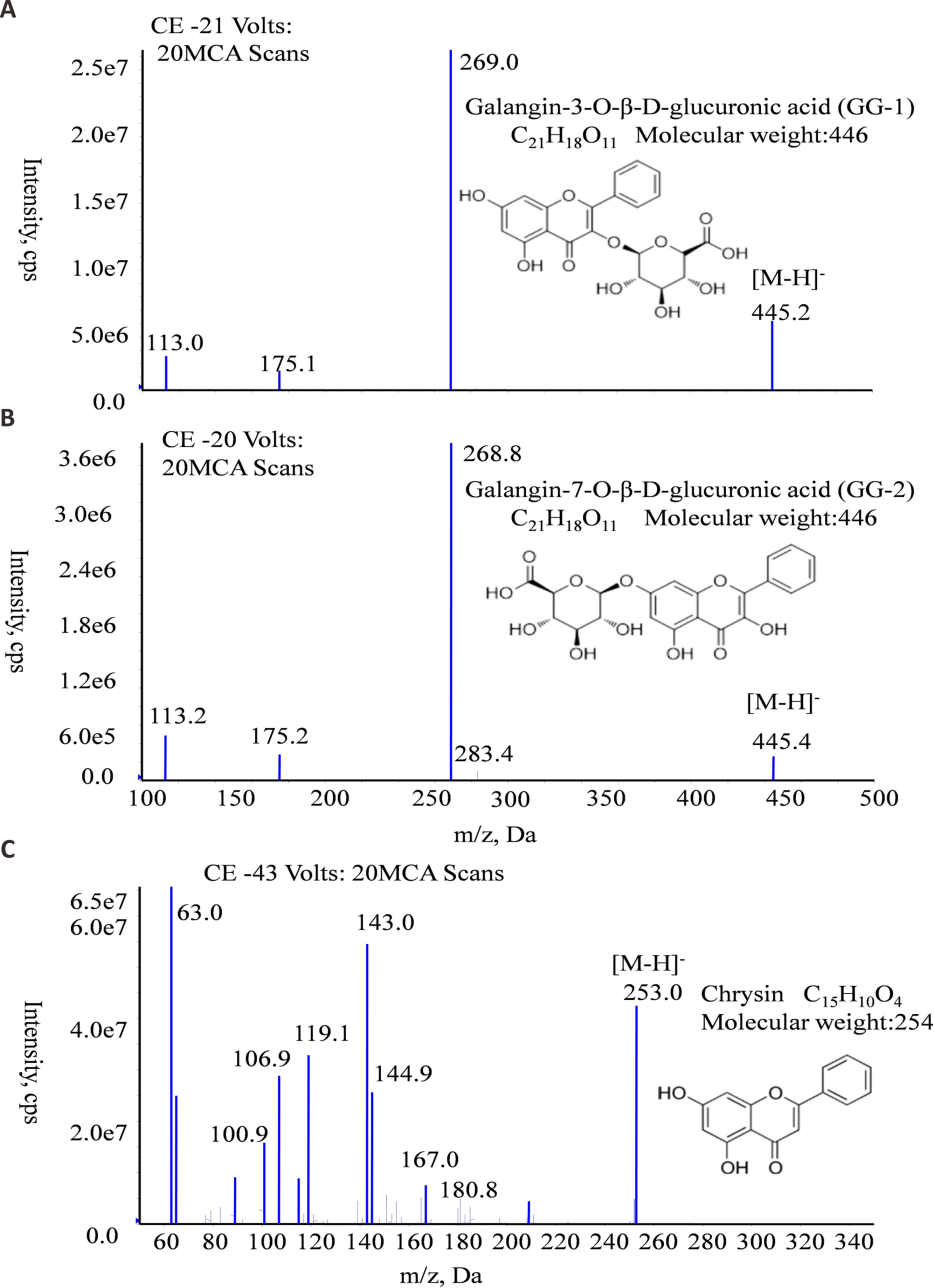
The molecular formula, molecular weight and MS/MS spectra of GG-1 (A), GG-2 (B) and chrysin (C).

**Figure 3:**
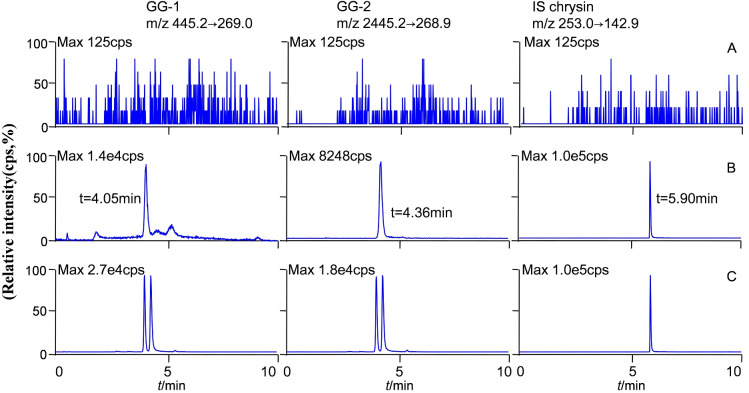
Representative chromatograms of GG-1, GG-2 and IS (chrysin) in different plasma samples after oral administration in rats. (A) Blank plasma samples; (B) standard plasma samples, 20 ng/mL; (C) plasma samples collected at 4 h.

The molecular formula, molecular weight and MS/MS spectra of GG-1, GG-2 and IS (chrysin) were shown in Fig. 2. The representative LC-MS/MS chromatograms of GG-1, GG-2 and IS are depicted in Fig. 3. The chromatographic peaks of GG-1, GG-2 and IS were eluted at 4.05, 4.36 and 5.90 min, respectively. GG-1, GG-2 and IS were well separated in the chromatograms. The results showed that the endogenous substances in rat plasma did not interfere with the detection of IS and the analytes.

#### Calibration curve and LLOQ

As shown in Table 1, the calibration curves were linear in the concentration range 2–2,000 ng/mL for GG-1 and GG-2. The average regression coefficients were 0.9976, 0.9982 for GG-1 and GG-2, respectively. The LLOQ for both GG-1 and GG-2 was two ng/mL.

#### Precision and accuracy

The intra- and inter-day precision and accuracy for QC samples are shown in Table 2. The intra- and inter-day precision (RSD) were varied from 2.1 to 12.8%, complying with the criteria that RSD must be less than 15%. As the bias ranged from −10.7 to +9.2% at all concentration levels, within the acceptance limits of ±  15%, which confirmed that the established method was accurate. All of the data confirmed that the analytical method was precise and accurate to quantitatively determine the plasma concentrations of GG-1 and GG-2 in rat plasma.

#### Matrix effect and extraction recovery

Matrix effect and extraction recovery data were shown in Table 3. The mean matrix effects calculated for GG-1, GG-2 and IS were in the range of 101.0–109.9% with RSD from 2.2–11.2%. Hence, ion variation in rat plasma was ignorable under the present experimental conditions. The mean extraction recovery for the analytes and IS varied from 89.1–101.8% with RSD ranging from 7.0–11.9%, proving to be precise and reproducible.

#### Stability

In this study, stability confirmation was carried out under various experimental conditions, i.e., short-term storage (4 h at room temperature), autosampler (8 h at room temperature), freeze-thaw (3 cycles) and 15-day stability. As shown in Table 4, RSD for GG-1 and GG-2 was not more than ± 15% at three different concentration levels. Therefore, the data suggested that the developed analytical method was stable under the above four conditions.

**Table 1:** The standard curve regression equation, linear range and LLOQ for GG-1 and GG-2 in rat plasma.

Analytes	Regression**equation (weighting, *r*^2^)	Linear range (ng/mL)	LLOQ (ng/mL)
GG-1	*y* = 0.0109*x* − 0.000342(1∕*x*, *r*^2^ = 0.9976)	2–2,000	2
GG-2	*y* = 0.00455*x* + 0.00624(1∕*x*, *r*^2^ = 0.9982)	2–2,000	2

#### Residual effect and dilution reliability

The residual peak area of the quantitative upper limit sample was less than 20% of the quantitative lower limit, and the residual peak area of IS was not more than 5% of IS peak area. The accuracy of diluted sample was within ± 15%, RSD <15%.

**Table 2:** The inter- and intra-day precision and accuracy for GG-1 and GG-2 in rat plasma (*n* = 6).

Nominal concentration (ng/mL)	Inter-day									Intra-day		
	Day 1			Day 2			Day 3					
	Measured (ng/mL)	RSD (%)	Accuracy (%)	Measured (ng/mL)	RSD (%)	Accuracy (%)	Measured (ng/mL)	RSD (%)	Accuracy (%)	Measured (ng/mL)	RSD (%)	Accuracy (%)
GG-1												
12	13.0 ± 0.8	6.4	109.2	11.7 ± 0.9	7.7	97.4	12.1 ± 1.6	12.8	100.8	12.3 ± 1.2	9.9	102.4
120	124.5 ± 9.5	7.6	103.7	112.5 ± 9.8	8.7	93.8	114.2 ± 8.3	7.2	95.0	117.1 ± 10.2	8.7	97.5
1200	1,087.0 ± 53.0	4.9	90.5	1,115.0 ± 60.0	5.3	93.0	1,152.0 ± 24.0	2.1	96.0	1,118.0 ± 53.0	4.7	93.1
GG-2												
12	12.7 ± 1.2	9.8	106.2	11.7 ± 0.8	6.5	97.4	11.7 ± 1.5	12.6	97.5	12.1 ± 1.2	9.8	100.7
120	128.8 ± 12.6	9.8	107.5	116.3 ± 10.8	9.3	96.8	111.7 ± 8.0	7.2	93.0	118.9 ± 12.5	10.5	99.1
1,200	1,072.0 ± 58.0	5.4	89.3	1,080.0 ± 80.0	7.4	90.0	1,126.0 ± 127.0	11.3	93.9	1,092.0 ± 90.0	8.3	91.1

### Application to pharmacokinetics study

**Figure 4:**
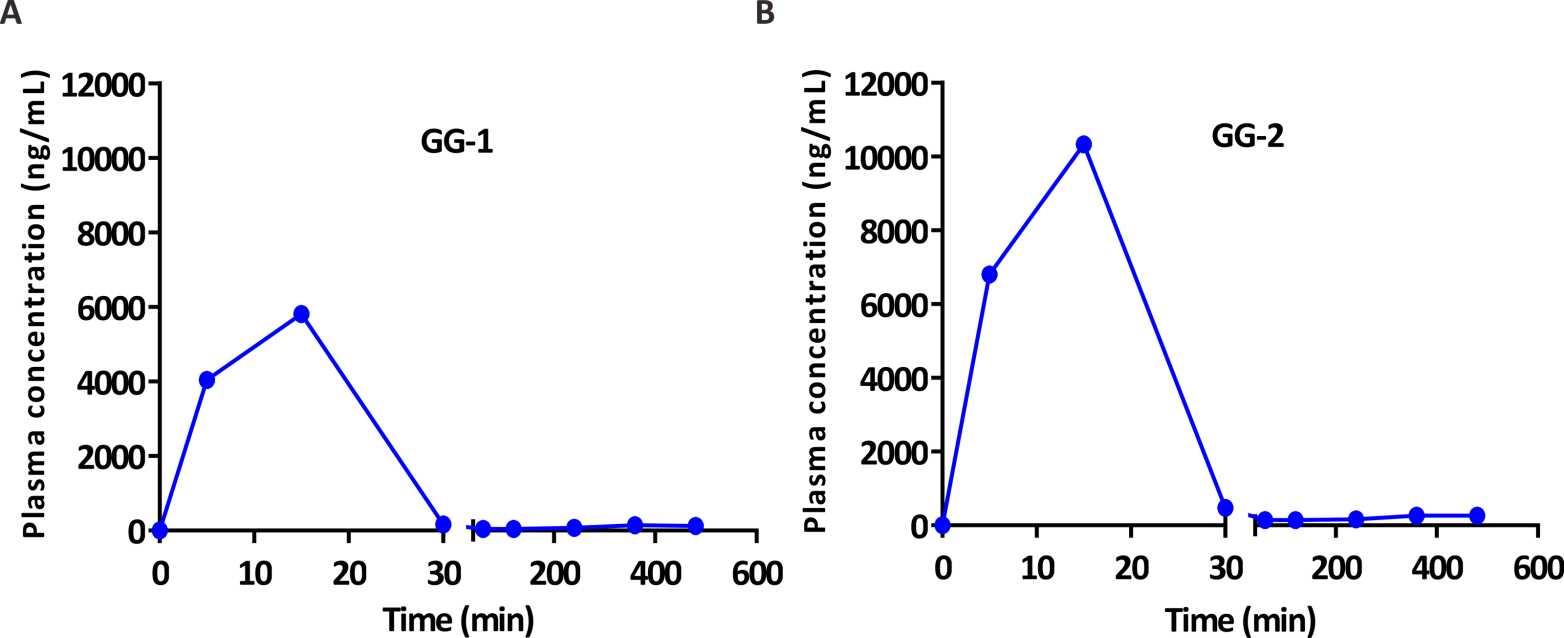
The plasma concentration-time curves of GG-1 (A) and GG-2 (B) after oral administration of galangal extract (0.3 g/kg) in rats.

After validation, UFLC-MS/MS analytical method was successfully applied in pharmacokinetic study of two galangin metabolites GG-1 and GG-2 in SD rats. The plasma concentration-time curves of GG-1 and GG-2 in rats after oral administration of galangal extract (0.3 g/kg) have been shown in Fig. 4. The pharmacokinetic parameters have been summarized in Table 5. C_max_ for GG-1 and GG-2 was 6069.6 ± 1140.6 and 10,596.0 ± 2395.7 ng/mL, respectively, and both achieved at 0.2 ± 0.1 h. The AUC_0−*t*_, MRT_0−*t*_, the elimination half-life (t_1∕2_) for GG-1 and GG-2 was found to be 2,390.9 ± 678.0 and 4,554.9 ± 884.9 h ⋅ ug/L, 1.4 ± 0.8 and 1.6 ± 0.7 h, 2.2 ± 0.7 and 3.3 ± 0.2 h, respectively. It should be pointed out that because the plasma concentrations of GG-1 and GG-2 at the blood collection points (12 h and 24 h) could not be detected, we only included the plasma concentrations from 0 h to 8 h after oral administration of galangal extract in the plasma concentration-time curves of GG-1 and GG-2.

**Table 3:** Matrix effect and extraction recovery for IS, GG-1 and GG-2 in rat plasma (*n* = 5).

Nominal concentration (ng/mL)	Matrix effect		Extraction efficiency	
	Mean (%)	RSD (%)	Mean (%)	RSD (%)
IS				
200	105.7	2.8	90.9	8.3
GG-1				
12	107.8	3.4	92.8	11.9
120	109.9	2.2	92.0	9.4
1200	101.0	9.3	101.8	7.4
GG-2				
12	101.9	4.8	100.6	7.0
120	108.4	3.0	95.0	8.8
1200	108.9	11.2	89.1	7.0

**Table 4:** Stability of GG-1 and GG-2 under four different experimental conditions in rat plasma (*n* = 5).

Experimental condition	Parameters	Nominal concentration (ng/mL)					
		GG-1			GG-2		
		12	120	1200	12	120	1200
Short-term stability (4 h at room temperature)	Mean ± SD	10.6 ± 0.6	113.4 ± 3.6	1,062.0 ± 76.0	12.0 ± 0.9	106.5 ± 7.7	1,062.0 ± 37.0
	RSD(%)	5.2	3.2	7.2	7.4	7.2	3.5
	Accuracy(%)	88.4	94.4	88.5	100.2	88.7	88.6
Autosampler stability (8 h at room temperature)	Mean ± SD	11.2 ± 0.6	109.4 ± 6.7	1,154.0 ± 131.0	10.8 ± 1.1	105.7 ± 8.0	1,064.0 ± 67.0
	RSD(%)	5.6	6.2	11.4	10.2	7.5	6.3
	Accuracy(%)	93.8	91.1	96.1	89.6	88.2	88.9
Freeze-thaw stability (3 cycles)	Mean ± SD	13.1 ± 1.0	109.3 ± 8.4	1,084.0 ± 32.0	12.8 ± 0.8	106.3 ± 6.3	1,122.0 ± 37.0
	RSD(%)	7.8	7.7	3	6.1	5.9	3.3
	Accuracy(%)	109.3	99.1	90.4	106.9	88.7	93.6
15 days at −20°C	Mean ± SD	11.1 ± 0.7	108.1 ± 3.5	1117.4 ± 73.2	11.4 ± 1.5	111.1 ± 6.1	1079.7 ± 31.7
	RSD(%)	6.4	3.2	6.6	12.7	5.5	2.9
	Accuracy(%)	92.5	90.1	93.1	95.5	92.6	90.0

## Discussion

Galangin is an important bioactive component of galangal which has been widely studied for the pharmacological activities. So far, there is little research focusing on galangin metabolism in vivo. Studies have proven that galangin could be metabolized to kaempferol and quercetin by cytochrome *P450* in rats (Hamada et al., 2010; Silva et al., 1997a; Silva et al., 1997b). As a flavonoid, once ingested, galangin undergoes various kinds of metabolism including glucuronidation, methylation and sulfation in the intestine and liver, leading to very low concentration of its original form in the body and an extremely low bioavailability (Thilakarathna & Rupasinghe, 2013). Glucuronidation is a major conjugation reaction that is catalysed by numerous UDP glucuronosyltransferase (UGT) isoforms, a group of phase II drug-metabolizing enzymes that are resident in the endoplasmic reticulum among which UGT1A3 and 1A9 are the main enzymes catalysing the glucuronidation of flavonoids in human liver (Green et al., 1998; Trubetskoy, Finel & Trubetskoy, 2008). Our previous research has also demonstrated that galangin was preferentially glucuronidated after oral administration but sulfated after intravenous injection medication (Chen et al., 2015). In this work, after oral administration of galangal extract in rats, we actually determined two galangin metabolites GG-1 and GG-2 which was in accordance with our previous conclusion that galangin was preferentially glucuronidated after oral administration. Besides, our previous study has indicated that both the glucuronidated and the sulfated were more efficient than free parent galangin which highlight the importance of conducting research on galangin metabolites (Zhang et al., 2016).

**Table 5:** Pharmacokinetic parameters for GG-1 and GG-2 after oral administration of galangal extract (0.3 g/kg) in rats (*n* = 5).

pharmacokinetic parameter	GG-1	GG-2
AUC_0−*t*_ (ug/L ⋅ h)	2,390.9 ± 678.0	4,554.9 ± 884.9
MRT_0−*t*_ (h)	1.4 ± 0.8	1.6 ± 0.7
*t*_1∕2_ (h)	2.2 ± 0.7	3.3 ± 0.2
T_max_ (h)	0.2 ± 0.1	0.2 ± 0.1
C_max_ (ug/L)	6,069.6 ± 1,140.6	10,596.0 ± 2,395.7

To preliminary study the bioavailability of two galangin metabolites GG-1 and GG-2, this work firstly developed an UFLC-MS/MS analytical method to simultaneously determine the two galangin metabolites in rat plasma in which calibration curve, LLOQ, precision, accuracy, matrix effect, extraction recovery, stability, residual effect and dilution reliability were all up to the FDA standard. Furthermore, we applied the validated method to acquire pharmacokinetic information for two galangin metabolitesGG-1 and GG-2 after oral administration of galangal extract in rats.As a result, we found that T_max_ of GG-1 was equivalent to that of GG-2, and MRT_0−*t*_, *t*_1∕2_ of GG-2 were a little higher than those of GG-1. The most significant differences between GG-1 and GG-2 were AUC_0−*t*_ and C_max_ in which the parameter values of GG-2 were almost twice as those of GG-1. To explain this phenomenon, we must learn that glucuronidation is affected by the binding sites of hydroxyl groups. Some studies focusing on structure—metabolism relationships of flavonoids reported that the preferred substrates for UGT1A3 and UGT1A9 contain the hydroxyl group at the C7-position (Xie et al., 2011). Additionally, the preferred substrates of UGT1A10 contain the hydroxyl group at the C7 position or C4′ of the B ring but not C5 of the A ring (Gan et al., 2020). Therefore, it is easy to understand that because the hydroxyl group at the C7-position of galangin is preferential to be glucuronidated by UGTs so AUC_0−*t*_ and C_max_ of galangin-7-O- β-D-glucuronic acid (GG-2) were much higher than those of galangin-3-O- β-D-glucuronic acid (GG-1).

On the other hand, in view that galangin possesses multiple biological activity, such as anti-cancer (Ha et al., 2013; Su et al., 2013; Zhang et al., 2013), anti-oxidative (Choi & Kim, 2014; Shu et al., 2014), anti-inflammatory (Jung et al., 2014; Kim, Bae & Kim, 2013) and lipid-lowering effects (Jung et al., 2012; Kumar & Alagawadi, 2013), and some studies have confirmed that glucuronidated metabolites of galangin have better bioactivities than parent galangin, we need to evaluate biological activities of galangin metabolites (chemically synthesized or separated from biosamples) to find more effective, safer, less toxic drugs from their active metabolites.

## Conclusion

This study developed an UFLC-MS/MS analytical method in order to simultaneously determine two galangin metabolitesGG-1 and GG-2 in rat plasma. Moreover, after validation we applied this analytical method to in vivo metabolism of galangin and acquired pharmacokinetic information including AUC_0−*t*_, MRT_0−*t*_, t_1∕2_, T_max_, C_max_ for GG-1 and GG-2after oral administration of galangal extract in rats. In short, this work provided a feasible analytical means and certain foundation for in vivo bioanalysis for galangin metabolites.

##  Supplemental Information

10.7717/peerj.11041/supp-1 Supplemental Information 1 Method validation All measured values in method validation.

10.7717/peerj.11041/supp-2 Supplemental Information 2 pharmacokinetic parameters of GG-1 The measured values of concentration-time and the calculated pharmacokinetic parameters of GG-1.

10.7717/peerj.11041/supp-3 Supplemental Information 3 Pharmacokinetic parameters of GG-2 The measured values of concentration-time and the calculated pharmacokinetic parameters of GG-2.

10.7717/peerj.11041/supp-4 Supplemental Information 4 Author Checklist-Full

## Additional Information and Declarations

### Data Availability

The following information was supplied regarding data availability: Raw data are available in the Supplemental Files.
